# Recommendations for developing and applying genetic tools to assess and manage biological invasions in marine ecosystems

**DOI:** 10.1016/j.marpol.2017.08.014

**Published:** 2017

**Authors:** John A. Darling, Bella S. Galil, Gary R. Carvalho, Marc Rius, Frédérique Viard, Stefano Piraino

**Affiliations:** a National Exposure Research Laboratory, United States Environmental Protection Agency, 109 T.W. Alexander Drive, Research Triangle Park, NC 27711, USA; b The Steinhardt Museum of Natural History, Israel National Center for Biodiversity Studies, Tel Aviv University, Tel Aviv 6997801, Israel; c School of Biological Sciences, University of Bangor, UK; d Ocean and Earth Science, National Oceanography Centre, University of Southampton, UK; e Centre for Ecological Genomics and Wildlife Conservation, University of Johannesburg, South Africa; f Sorbonne Université, Université Paris 06, CNRS, UMR 7144 AD2M, Station Biologique de Roscoff, Place Georges Teissier, 29680 Roscoff, France; g Dipartimento di Scienze e Tecnologie Biologiche ed Ambientali, Università del Salento, Lecce, Italy; h Consorzio Nazionale Interuniversitario per le Scienze del Mare (CoNISMa), Roma, Italy

**Keywords:** Marine invasive species, Surveillance, Monitoring, Early detection, Environmental DNA, Metabarcoding, Good environmental status, High throughput sequencing, Marine Strategy Framework Directive

## Abstract

The European Union’s Marine Strategy Framework Directive (MSFD) aims to adopt integrated ecosystem management approaches to achieve or maintain “Good Environmental Status” for marine waters, habitats and resources, including mitigation of the negative effects of non-indigenous species (NIS). The Directive further seeks to promote broadly standardized monitoring efforts and assessment of temporal trends in marine ecosystem condition, incorporating metrics describing the distribution and impacts of NIS. Accomplishing these goals will require application of advanced tools for NIS surveillance and risk assessment, particularly given known challenges associated with surveying and monitoring with traditional methods. In the past decade, a host of methods based on nucleic acids (DNA and RNA) analysis have been developed or advanced that promise to dramatically enhance capacity in assessing and managing NIS. However, ensuring that these rapidly evolving approaches remain accessible and responsive to the needs of resource managers remains a challenge. This paper provides recommendations for future development of these genetic tools for assessment and management of NIS in marine systems, within the context of the explicit requirements of the MSFD. Issues considered include technological innovation, methodological standardization, data sharing and collaboration, and the critical importance of shared foundational resources, particularly integrated taxonomic expertise. Though the recommendations offered here are not exhaustive, they provide a basis for future intentional (and international) collaborative development of a genetic toolkit for NIS research, capable of fulfilling the immediate and long term goals of marine ecosystem and resource conservation.

## 1. Introduction

The introduction of non-indigenous species (NIS) represents a major driver of ecological and evolutionary change in the world’s oceans, often resulting in dramatic restructuring of biotic communities [1,2] and shifts in ecosystem function with impacts on the availability of marine resources and ecosystem services [3,4]. Like their counterparts in terrestrial and freshwater systems, marine biological invasions continue to be driven by pressures associated with human activity and global trade [5], and their spread at multiple spatial scales remains tied to the increasing activity of anthropogenic vectors of species introductions such as vessels, aquaculture, interoceanic canals and aquarium trade [6–10]. This ongoing shuffling of marine biodiversity occurs in the context of multiple other anthropogenic stressors, resulting in significant challenges to the sustainable management of marine resources, particularly in coastal environments [11].

Recognition of these challenges has led to the creation of policies, conventions, and various other legislative frameworks aimed at preventing future introductions and mitigating or reversing the impacts of existing marine invasions. One of these is the European Union Marine Strategy Framework Directive (MSFD, Directive 2008/56/EC [12]), which aims to adopt integrated ecosystem-based management approaches to achieve or maintain “Good Environmental Status” (GES) for marine waters, habitats and associated resources, including the goal that “non-indigenous species introduced by human activities are at levels that do not adversely alter the ecosystem” (MSFD, Descriptor 2). Among the various objectives of the MSFD is the promotion of regionally standardized monitoring approaches capable of delivering temporal datasets reflecting long-term trends in marine ecosystem status, incorporating indicators related to the abundance, distribution, and impacts of NIS.

NIS are often challenging to monitor using traditional field survey and identification methods. This is especially true at early stages when NIS are rare, hampering detections of incipient invasions. In addition, these methods are often ineffective for identifying NIS lacking diagnostic features, such as larval stages—an essential contributor to propagule pressure. The same holds for complex microscopic taxa or taxa that are not easily sampled (e.g. in circalittoral rocky substrates, in sediments, etc.). Further, the reliable identification of NIS depends on expert taxonomic knowledge at a global and regional level, which is unfortunately decreasing (see Section 2.1 for further discussion). These challenges may explain in part why progress on NIS monitoring lags considerably behind other efforts aimed at achieving GES among MSFD Member States [13]. They also explain recent recognition of genetic approaches for monitoring, preventing, and managing biological invasions in marine systems. For instance, in response to the stated ambitions of the MSFD members of the International Convention for the Exploration of the Sea (ICES) Working Group on Introductions and Transfers of Marine Organisms (WGITMO) recently outlined 10 key requirements for NIS assessment and management [14], including the development and application of genetic tools. Technical guidance on MSFD monitoring initiatives have similarly recommended development of routine molecular methods [15], and advancement of molecular tools have been recognized more broadly as general emerging priorities for invasion science [16]. Already, molecular methods for targeted species detection and community profiling are being broadly applied in the context of marine biodiversity monitoring [17], and additional approaches such as reconstruction of invasion histories and analysis of rapid evolutionary change in the context of invasion risk assessment present novel avenues of research with significant potential application in decision-making contexts. Rapid technological advances, including dramatic increases in cost-effectiveness [18–20], continue to render such tools increasingly attractive to a wide range of potential end-users.

Nevertheless, it is clear that existing molecular genetic tools could be made more responsive and relevant to management needs [21]. In particular, progress must be made toward standardized approaches in order to accomplish the harmonization of global and regional long-term datasets. This need is made explicit in recent EU decision 2017/848 [22], which outlines the importance of “specifications and standardised methods for monitoring and assessment,” including the definition of threshold values. For NIS, recommended criteria and methodological standards for GES assessment require Member States to establish a) regional and subregional inventories of NIS, b) the number of new introductions over a 6-year assessment basis, and the definition of a threshold value; c) abundance and spatial distribution of NIS and particularly of invasive species, and their adverse effects on native species groups and habitat types. These efforts will require not only improvements in genetic surveillance methods, but also better integration of molecular approaches with traditional methods and more effective communication of the outcomes of molecular research and surveillance. Such steps would ultimately contribute to more accurate, transparent, and cost-effective assessments of the distribution and impacts of NIS in marine systems.

## 2. Recommendations for developing and applying molecular tools

This article expands considerably upon previous recommendations for developing and applying molecular tools [14], identifying specific goals that would effectively speed the development and implementation of molecular approaches for NIS management with the aim of achieving the stated objectives of the MSFD. It explores seven areas of critical importance for advancing the utility of molecular tools for NIS research and management in marine systems. These issues can be roughly categorized on the basis of the appropriate time horizon for action. Recommendations 2.1 through 2.3 represent research needs that should be addressed immediately, largely in order to improve the utility of genetic tools that are already being or soon to be adopted in monitoring contexts. 2.4 and 2.5 are recommendations for shifts in research focus that will likely require concerted action on near- to mid-term timeframes. Items 2.6 and 2.7 are anticipatory recommendations, recognizing the likelihood of future technological developments that have not yet been realized but that could have dramatic implications for long-term NIS management.

### 2.1. Investment in improved taxonomic resources and support for integrative taxonomy

Ojaveer, et al. [14] have already highlighted the availability of taxonomic expertise as a critical general requirement for NIS assessment and management, and that argument bears repeating in the specific context of molecular tool development and implementation. The dramatic rise of **DNA barcoding** (see Box 1 for definitions of bolded terms) and related approaches to biodiversity assessment has served to emphasize the dependence of these molecular methods on traditional taxonomic knowledge. DNA barcoding is an easy-to use, cost-effective and standard approach useful to validate species identification based on morphological traits (Table 1). But to be effective and accurate, each taxonomically accepted species has to be identified with a specific barcode (i.e. a specific molecular reference). DNA sequence data is therefore often only as good as the **reference database** used to interpret it, and those databases in turn are only as good as the taxonomic expertise behind deposited reference sequences and associated meta-data [23,24]. Unfortunately, such expertise has been in decline for decades [25,26]. This loss raises the specter of crippling bottlenecks to advancement of our understanding of marine biological invasions and marine biodiversity. Already a proliferation of molecular studies indicates the likelihood of previously unrecognized cryptic diversity, resulting in numerous systematic hypotheses that in many instances remain untested through integrated taxonomic study [20,27]. The failure to resolve these issues can lead to confusion regarding both invasion history and the degree to which marine biota have been shaped by species introductions [28,29].

The emergence of **high throughput sequencing (HTS)** and its application to biodiversity studies through **metabarcoding** [30] further underlines the critical importance of renewed investment in traditional taxonomy. Metabarcoding introduces the possibility of **non-targeted surveillance** of molecular biodiversity landscapes, in which whole communities can be explored without prior specification of monitoring targets (by extracting and barcoding either **bulk DNA** from environmental samples or **environmental DNA** (eDNA; see Section 2.3 below and Table 1)), and further presents novel opportunities for incorporating into biodiversity monitoring certain speciose and ecologically important groups that still remain understudied by traditional taxonomic means, such as eukaryotic meiofauna [31,32]. While metabarcoding efforts may begin to hint at the diversity of such organisms in marine systems, and at the extent to which regional meiofaunal biota have been shuffled by human activity, without dedicated taxonomic investigation these hints will largely be missing ecological and evolutionary context. Concerted investment in taxonomic resources should aim to move beyond “classical” morphological taxonomy and toward development of expertise in **integrated taxonomic assessment** (assimilating insights from multiple disciplines, including molecular taxonomy), and should encourage progress toward collaborative, readily accessible biodiversity information systems tailored to the needs of managers and decision-makers [33]. A handful of recent studies suggests that integrated taxonomic approaches offer powerful tools for clarifying important questions of species identity and invasion history [34–37], with associated implications for NIS management.

Of particular relevance to the development and application of molecular tools is the curation of accurate, appropriately vouchered DNA sequence data, along with accessible long-term depositories for associated source specimens. In this respect, substantial resource investment in networks of marine stations (such as the European Marine Biological Resource Centre, EMBRC) might help to build focal points of observatory NIS networks, serving as repositories for vouchered specimens, DNA/tissue samples, and taxonomic expertise, all while leveraging existing collections and their potential value for untangling invasion histories [38]. One of the greatest challenges for metabarcoding studies is the lack of reference sequences for determination of species identities based on barcode data. While the growth of dedicated bar-coding databases maintaining stringent quality control standards (e.g. the Barcode of Life Database, http://www.boldsystems.org/) has proceeded rapidly, such repositories still barely scratch the surface of estimated extant eukaryotic diversity, particularly in marine systems [39], and are heavily biased toward taxa and regions that have been well sampled and receive generous support for molecular data collection [23,40,41]. With a few exceptions (e.g [42].), there have thus far been a limited number of initiatives aimed directly at building up molecular reference data dedicated to NIS. Although the usefulness of barcoding studies to develop marine NIS inventories is already well documented [43], building up a dedicated, regularly updated, accurate reference database is a cornerstone to sustain efficient NIS molecular (meta)barcoding approach, and promoting such an initiative at the European level would clearly support MSFD objectives. Fortunately, evidence suggests that focused dedication of human and financial resources can result in rapid accumulation of expertly curated taxonomic information relevant to broad scale metabarcoding efforts [44].

### 2.2. Standardization of protocols for generating molecular genetic data and development of user-friendly data analyses workflows

To most effectively satisfy the aims of the MSFD, methods should be broadly applicable and should generate data that are auditable, easily communicated, and comparable across studies conducted by multiple research groups and management bodies in multiple regions. Molecular data have the potential to satisfy these criteria in ways that may have advantages over traditional morphological approaches. For instance, molecular data allows highly objective comparison of analytical results across studies [45], and the growing information infrastructure devoted to molecular data provides unique opportunities to make results publicly available, and to attach those results to existing biodiversity information networks. This, along with the ease with which DNA can be archived almost indefinitely, also facilitates re-analysis and objective comparisons of biodiversity estimates across longitudinal studies. In addition, comparisons between traditional and metabarcoding-based biodiversity surveys suggest that the latter is likely to be cost-effective, requiring far less investment in human resources and expertise [46]. This cost differential is only likely to broaden as the generation of sequence data becomes increasingly less expensive and access to molecular infrastructure and expertise becomes more common.

Realizing the full promise of molecular tools, however, will require concerted effort to standardize methodologies and data handling across broad networks of researchers and resource managers. For instance, **targeted surveillance** of target species via application of probe-based molecular methods (e.g. PCR, qPCR) is efficient for targeting NIS and can enable highly sensitive delineation of population distributions in aquatic systems [47,48] (Table 1). However, the utility of this approach, particularly if implemented at broad spatial scales, is contingent on adoption of standard practices ranging from field sampling protocols to laboratory quality assurance standards and selection of appropriate target loci. The molecular resources required to develop targeted detection tools are widely available, and in many cases the investigators pursuing such development may be unfamiliar with or unprepared for the scrutiny that accompanies application of detection tools in decision-making contexts [49]. Formulation and dissemination of guidance for the development of targeted molecular detection methods would greatly facilitate the transition of new technologies from the laboratory to field application. That guidance should include widely accepted protocols for sampling (e.g. sample preservation, handling, and the use of field controls) and laboratory quality assurance as well as standards for determination of error rates associated with molecular detections, i.e. sensitivity and specificity under known environmental parameters. It should be noted that while the focus here is on protocols associated with molecular workflows, the issue of sampling design is a critical one and likely to have just as much influence on the value of surveillance efforts as the quality of molecular data [50]. That issue is not unique to molecular genetic surveillance, although there may be considerations that are particularly challenging in this context (see Section 2.3 below). Given differences across taxa in the usefulness of potential genomic targets for probe design [51], it is unlikely that specific recommendations for standardizing such design can be offered *a priori*. However, identification of best practices for *in silico* design of molecular probes and laboratory testing for sensitivity and specificity should be achievable. Furthermore, support for data and information sharing could dramatically speed method development, and formal early adoption of effective tools could ensure that results of surveillance conducted across multiple studies are directly comparable.

Similar considerations should be applied to community profiling based on HTS data. Metabarcoding workflows are complex, with a large number of processing steps standing between an environmental sample and information that may be useful to decision-makers. The options available at each processing step can result in substantial variation between studies [52], as each tailors its workflow to the needs of a particular application. Unfortunately, this variation can lead to analytical results that are uncomparable across studies, presenting a significant challenge for any distributed monitoring network that hopes to rely on metabarcoding data. Recent work has demonstrated that choices in HTS data processing can dramatically influence analytical outcomes, and numerous studies have revealed the dependence of diversity assessments on the choice of barcode locus [53–56].

Standardization of early steps in metabarcoding workflows (i.e. sample processing and DNA extraction) should ideally be harmonized with applications that may rely on the same samples (e.g. targeted DNA-based monitoring based on quantitative PCR or other methods). Fortunately, a significant literature exists assessing the utility of various protocols for sample handling appropriate for molecular tasks [57–63]. Adoption of standards for barcoding loci may present greater challenges. A number of studies have evaluated the utility of specific primers and/or loci to encompass a wide set of taxa [55,56,64–66]. As there is no universal barcode, the development and validation of primers designed specifically for targeted taxonomic groups greatly increases both detection probability and accuracy of taxonomic assignment [67]; such efforts may include attempts to assess the versatility of selected primers for describing known species assemblages acting as “living voucher repositories,” such as at aquarium facilities [68]. However, given decreasing HTS costs and increasing throughput as well as read length it is more and more feasible to incorporate analyses based on multiple evolutionarily independent barcode loci into single studies [52,55]. Ultimately, sequencing multiple and specific loci would not only increase taxonomic breadth, but would enable novel error identification approaches based on comparison of results across loci.

Another significant challenge will be development of standardized analytical pipelines. Options for sequence data processing are myriad, with dozens of algorithms available that are rapidly evolving [31,53]. Ideally, determination of the most appropriate analytical pipeline would require experimental evaluation of the implications of pipeline design for downstream applications [53]. Given the vast number of potential combinations of options across multiple steps of the HTS workflow, design of standard analytical protocols will likely fall to expert opinion based on existing comparative studies, with an eye to generation of information most useful to end-users. Of special importance will be the question of stringency. Concern for the possibility of sequence error has led to workflows that remove very rare sequences (particularly singletons) from downstream analysis. Unfortunately, recent work has demonstrated that the resulting estimates of biodiversity are conservative and may fail to detect rare species that are truly present in the sample [54,69]. Designers of bioinformatics pipelines may be forced to decide whether the importance of detecting rare species (in order to register, for instance, incipient invasions) may require more liberal interpretations of the data via inclusion of rare sequences. Generally, analytical protocols should be developed with the explicit goal of robustness and consistency across sampling sites throughout the monitored region and across an appropriate monitoring time frame. The latter will entail careful consideration of recent and likely future developments in HTS platforms, to ensure that analytical approaches can remain relevant in the face of rapidly changing technology. The key challenge is to provide guidance on standard protocols that will both enable inter-site comparisons of HTS-produced data and ensure sufficient confidence in analytical results to support management and policy decisions. Development of unified guidance on such standardization would be a significant step toward improving the transparency and utility of data generated by molecular surveillance tools. Moreover, the prerequisite for standardization in sample details and methodology promotes comparability and accessibility of such data sets for archiving.

Thus far, interpretation of molecular genetic surveillance data has generally required considerable technical expertise, particularly in bioinformatics. Efforts to achieve standardization of protocols will involve development of user-friendly pipelines accessible to the nonspecialist. This could be rapidly achieved for straightforward targeted surveillance, such as monitoring for specific NIS for which molecular reference is available. Bioinformatic experts could be supported to develop tools that can be handled by both specialists and non-specialists, for instance by offering options appropriate to varying levels of end-user expertise. Such a strategy has been adopted for other statistical molecular analyses, for example for phylogenetic studies (e.g. Phylogeny.fr, a suite of programs with a web based interface offering three levels, ‘one-click’, ‘advanced’ and ‘a la carte’ mode, each of them targeting a specific public [70]). For most applications targeting complex biotic communities, given the vast number of potential combinations of options across multiple steps of the HTS workflow, development of such tools remains a promising but longer-term effort.

### 2.3. Reduce uncertainty associated with inferences of NIS distributions based on molecular detections

One of the most obvious applications of molecular tools for the management of marine bioinvasions is the delineation of NIS distributions (Table 1). Understanding those distributions is critically important not only to enable assessment of risks posed by known introduced species (particularly new incursions of potentially invasive populations), but in many cases simply to identify which taxa have been introduced and where. Recent efforts to delineate species distributions have been dramatically altered by the possibility of coupling molecular genetic detection methods with eDNA analysis, in which organismal DNA is collected without any attempt to capture the target organism itself. This approach greatly simplifies sample collection, allowing rapid processing of very large numbers of samples and thus encompassing many putative introduction localities, seasons and habitats. The use of eDNA based surveys might be particularly effective in those situations poorly suited to traditional surveillance efforts, e.g. detection of species in remote locations, inaccessible or under-studied habitats or communities, detection of incipient invasions or moving invasion fronts, or monitoring of presumed failed or eradicated populations. Efforts to determine NIS distributions directly support development of indicators associated with MSFD Descriptor 2, particularly those that rely on accurate assessment of trends (2.1.1, 2.2.1). Given recognized limitations of traditional survey approaches, DNA-based methods will likely be critically important components of future surveillance toolkits [20].

The advent of eDNA-based surveillance has elicited not only recognition of the possibility of “sight-unseen” detection of target species, but also realization of the potential uncertainties associated with such detections [71–73]. Most obviously: If DNA can be detected in the absence of the target organism, then how can the presence of that organism be inferred confidently from the presence of its DNA? This question still poses clear challenges in decision-making contexts (e.g. rapid response to novel incursions) and more generally for the inference of biodiversity trends derived from molecular surveillance data. Fortunately, a number of recent studies have made advances toward understanding the complex relationship between patterns in detection of DNA and the underlying distributions of organisms associated with that DNA. These advances suggest that future investment should bring increasingly robust methods for interpreting molecular genetic surveillance data, and greater utility for the assessment and management of marine bioinvasions. Interactions between extraorganismal DNA and the various environmental factors that determine its dispersal and persistence in aquatic systems have been explored [74–76], as well as the degree to which molecular data might enable inference of specific characteristics of target populations—not only species identities, but also estimates of organism abundance and their distribution in time and space [77–79]. It is thus becoming increasingly clear that DNA-based monitoring can provide ecologically relevant information with significant potential for informing to the management of marine resources [80].

These developments presage more sophisticated applications of molecular genetic surveillance data. While simple maps of positive and negative detections may be of some use to managers, in the context of continuous monitoring of population trends they are perhaps more appropriately thought of as un-interpreted raw data. Of considerably greater utility would be models that estimate the likelihood of target population distributions based on observed detection patterns and known rates of false positive and negative errors, as well as other influencing environmental factors. A growing literature attests to the possibility of generating such models, which offer not only to interpret raw presence/absence data for downstream users, but also to communicate associated uncertainties. Most promising is the development and evaluation of **site occupancy-detection modelling (SODM)** frameworks for translating eDNA-based detections into management-relevant estimates of target population distribution [81–83]. A host of recommendations for molecular monitoring best practices have emerged from this literature, including rigorous laboratory determination of error rates, increases in replication, incorporation of prior information or collection of additional non-molecular sampling data, and application of appropriate statistical frameworks for model development. Early indications are that adoption of such practices can dramatically reduce the risk of false positive detections, increase the per-sample information value of monitoring efforts, and provide more robust and reliable inferences of target species abundance and distribution. The guidance emerging from these studies should provide a foundation for implementation of eDNA-based monitoring efforts in the context of MSFD trends assessment. Future refinement of SODM approaches, along with growing understanding of the environmental factors impacting DNA persistence in marine systems, should enable the development of clear recommendations for molecular monitoring practices aimed at understanding the distributions of NIS in European seas. More complete understanding of the uncertainties associated with these approaches should allow more sophisticated design of overall surveillance programs that couple molecular genetic and traditional detection methods, taking advantage of the relative strengths and weaknesses of each.

### 2.4. Support for coordinated species-specific research activity

Detailed molecular genetic analysis of single species can often provide important insights into historical patterns of invasion and future risk of population expansion or impact. Application of analytical approaches in the fields of population genetics, evolutionary ecology, and genomics have dramatically reshaped understanding of biological invasions [84], and in marine systems a host of recent studies attest to the value of these methods for unraveling issues of taxonomic identity, invasive origins, population dynamics, and evolutionary trajectories [19,20,27].

Development of appropriate high resolution molecular markers is a costly prerequisite for robust reconstructions of complex invasion histories [18] or understanding the putative impacts of new introductions (e.g. hybridization with native species [85]), as are intensive sampling efforts in both source and recipient regions [86]. Investigation of the evolutionary consequences of invasion (e.g. adaptation of introduced populations to novel selection pressures) similarly requires development of molecular resources (genomes, transcriptomes, or large panels of variable markers) that necessitate substantial investment [87]. As a result, taxa are frequently chosen for detailed investigation based on either prior availability of such resources or idiosyncratic interests of independent research projects, and not necessarily on criteria directly relevant to managers or other decision-makers.

*A priori* identification of promising targets for detailed molecular analysis could result in unprecedented opportunities to leverage limited resources efficiently. Currently effort is broadly distributed across an extraordinary diversity of taxa, the result being that many studies are too resource-limited to generate results robust enough to influence management [21,88]. Greater focus on a suite of species identified by cooperative agreement between molecular ecologists and resource managers may address this shortcoming, and could substantially increase the likelihood of generating information with direct value for decision-makers. In addition to providing insights into population characteristics with potential relevance to invasion risk (e.g. likely invasion corridors and vectors, possibility of adaptive response to novel stressors including climate change, population-specific niche requirements for assessing likelihood of further spread, etc.), coordinated molecular investigation could also generate the knowledge required to weigh the risks and benefits of advanced control methodologies (see Section 2.7, below).

Identification of taxa for prioritization is neither a simple nor a novel task. Lists of marine species targeted for prevention or other forms of management have been developed at national and/or regional scales [42], and can be based on a wide range of possible criteria including invasion risk and likelihood of ecological and economic impact. Identification of additional NIS deserving of concerted attention under the auspices of the MSFD could be a challenging undertaking, but one with clearly defined benefits in terms of molecular genetic analysis. Coordinated funding could encourage sharing of molecular resources across coalitions of researchers, establishment of banks to house properly preserved specimens and tissues collected by widespread communities of scientists and managers, and communication of expertise, including application of state-of-science statistical analytical tools [18,19].

This strategy brings with it myriad challenges associated with data sharing and the conduct and publishing of research, but such challenges are not unprecedented and have been addressed successfully in other disciplines adopting similar coordinated approaches to large-scale data generation, most notably in the field of genomics [89]. Identification of appropriate taxa for targeted study requires collaboration between molecular geneticists, managers, organismal biologists and ecologists, and even those with appropriate expertise in assessing socio-economic risks. These skillsets will be crucially important for effective horizon scanning, to determine which NIS are both most likely to impose substantial costs (ecologically and economically) and also most likely to be amenable to intensive molecular investigation. Such a strategy has the additional benefit of supporting other coordinated actions referenced in sections above, for instance, building up in a coordinated way accurate and updated reference data targeting NIS, or promoting the development of shared collections of vouchers, DNA, and environmental samples.

### 2.5. Emphasize broader understanding of invasion pathways through community genetics

Invasion biology has focused largely on populations and species, and vectors of introduction have been traditionally thought of primarily as conveyances for those entities. In reality, introduction events typically entail the release of complex assemblages of organisms, if not entire functionally integrated communities. Molecular tools, particularly the rapidly advancing field of community metabarcoding, should enable the next generation of invasion biologists to seek a more holistic understanding of the ecology of invasion events and the role of particular pathways not simply as narrow conduits for NIS, but as broad corridors connecting continuously changing ecological systems. This perspective may alter not only ways of thinking about the risks and impacts of marine bioinvasions, but also ways of thinking about how they are managed.

The insight that vectors can transport species assemblages is not new. For instance, hull fouling has long been recognized as a vector capable of translocating complex biotic communities intact even over lengthy voyages [90], and the role of “colonization pressure” (the number of species transported) in determining invasion risk has been generally acknowledged [91]. However, molecular tools have the potential to expand these insights dramatically by enabling characterization of taxa not readily amenable to traditional morphological assessments. While previous studies have focused, necessarily, on the macrobiotic component of vectored species assemblages, emerging molecular tools allow investigation of a wide variety of meio- and microfaunal constituents, including biofilms, bacterial communities, viruses, protozoans and larvae [92–96]. Recent applications of **metagenomics**, for instance, have demonstrated the utility of these approaches for providing more comprehensive appraisals of meiofaunal bioinvasions in terrestrial systems [97]. Assessment of these community components could be critically important to understanding the full risks posed by introduction events. Some studies suggest that introducing co-evolved species complexes may have significant influence on the impact of invasions in marine systems [98]. This is particularly evident in cases of NIS and their associated parasites and other symbionts [99,100]. More comprehensive characterization of introduced biotic communities, facilitated by broader application of metabarcoding methods, may reveal how widespread such co-introductions are in marine environments and provide a stronger scientific basis for assessing potential impacts or tracing invasion histories. Perhaps more importantly, the ability to describe microbiota could herald a new paradigm in understanding the role of introduction vectors in changing ecological relationships in our seas, with associated implications for understanding trends in environmental status of marine resources. The critical importance of microbial ecology for both organismal health and ecosystem function, and the growing appreciation for the role of marine microbial networks in driving overall ecosystem dynamics [101,102] suggest that shifts in microbial community structure associated with biological invasions could have dramatic unseen consequences [103]. Metagenomic analyses of introduced holobionts (host organisms plus their associated microbiomes) could provide novel insights not only into invasion history, but also into the post-introduction fates of invaders and thus their potential for ecological impact [104].

Support for future research should cultivate this new perspective by encouraging the application of molecular tools to characterization of non-traditional communities associated with well-known vectors of introduction, particularly vectors such as ballast water, hull fouling, and shellfish culture known to deliver complex biotic assemblages. The assumption is that researchers and managers have been considering only a small part of a complex phenomenon in terms of the role that these vectors play in connecting regional biota. Many of the aforementioned recommendations apply similarly here—investment in foundational intellectual resources and development of standardized protocols for community assessments will be critically important to the success of this research program.

Also of potential interest may be the advancement of “taxonomy-free” approaches for interpreting metabarcoding data [30]. These methods, in which **operational taxonomic units** are adopted as the fundamental data of biodiversity assessment without attempt to attach known species identifications, have been employed for decades by microbial ecologists. It is possible that such taxonomy-free HTS-data analyses could serve as fingerprints of community diversity located in time and space, and that comparing fingerprints could provide novel insights into trends in ecological status and assessment of impacts of human activities [105], or even serve as indicators of biotic connectivity driven by anthropogenic vectors (Table 1). Such methods may become more accessible—and possibly more necessary—with increasing ability to rapidly and inexpensively generate sequence data.

### 2.6. Encourage development of novel in situ and field-deployable detection platforms

Technological advances in nucleic acids analysis have entailed not only increased throughput and decreasing costs, but also considerable reductions in the physical footprint of analytical apparatus. The movement in medicine and public health to leverage these advances for point-of-care diagnostics [106] has been paralleled by a similar movement to design in situ or field-deployable detection platforms for environmental monitoring. Innovations in microfluidics, combined with development of methods for highly sensitive detection of target nucleic acids that obviate lengthy PCR cycles, promise to yield continued progress toward these goals [107]. Growing automation and miniaturization of molecular workflows has also allowed development of robotic monitoring platforms capable of collecting real time in situ data on the distribution of marine organisms, including bacteria, phytoplankton, and zooplankton [108,109]. The “ecogenomic” data collected by these tools can be obtained at extremely high resolution, can link molecular detections with associated information on environmental condition and water quality, and can be controlled remotely by adaptive algorithms that allow observations targeted in both time and space [110].

The potential benefits of such tools for marine environmental monitoring are vast [111] and applications for detection of NIS are being actively explored, with some platforms exhibiting promise for challenging tasks such as real-time monitoring of ballast water samples [112]. These tools thus have potential not only to improve assessments of NIS distributions and trends in diversity (native and non-native) critical to appraisal of environmental condition, but also to enable management-relevant early detections of high profile marine invaders. The latter possibility suggests not only that future tools might allow rapid responses to incipient invasions or even preventative interdictions of contaminated vectors, but also raises the important issue of rigorous validation required to ensure reliability of detections in those contexts. Encouragement of further innovations in this field should thus be coupled with guidance to researchers on best practices for achieving standards of reliability appropriate to the expected end use of the technology. Such standards will be particularly important to ensure consistent application of tools and interpretation of data across different platforms deployed in different systems. One possible mechanism to address this issue would be development of technology verification programs capable of issuing type approvals for in situ tools that are fit for purpose. Such programs not only provide a means to ensure reliability of novel technologies, but also can help insulate investors in research and development from uncertainty.

### 2.7. Provide guidance on the development and application of advanced biotechnologies for NIS control

When prevention fails, control and/or eradication may be the remaining options for mitigating the impacts of invasive marine populations, when protection of native biodiversity and environmental condition is the objective. Besides ecological, social and ethical issues regarding eradication strategies [113], these approaches have always presented scientific challenges, and successful eradications have generally been highly localized and assisted by naturally limited connectivity between highly urbanized environments and surrounding systems; even so, they are undertaken only at considerable expense [114,115]. Control and eradication of marine NIS established in more open natural environments may be impossible with existing technologies, particularly given typical delays between establishment and discovery [116]. Efforts to control marine NIS are hampered not only by restricted accessibility, difficulties associated with targeted applications, and dispersal mechanisms peculiar to marine systems [113], but also by high risks associated with the kinds of classical biological control methods that have exhibited effectiveness in terrestrial systems [117]. These considerations have encouraged exploration of genetic control options for marine and other aquatic invaders. Methods such as release of sterile triploids, gender distortion, and Trojan genes, for example, have been investigated extensively for potential control of fish populations [118].

Novel developments in genomic modification have ushered in a paradigm shift in such genetic biocontrol technologies. The discovery and development of easily manipulable and evolutionarily stable selfish genetic elements, most notably those based on the **CRISPR-Cas9** system, now present radically new possibilities for genetic alteration of wild populations, including potential solutions to the problems of control and eradication [119,120]. These elements bias inheritance by inducing cells to copy them into homologous chromosomes, thus insuring that they are passed to all offspring of sexual reproduction. In so doing, they provide the basis for **gene drives** that enable the rapid spread of traits through wild populations with short generation times—even if those traits have been engineered and would under normal circumstances be eliminated by natural selection [119]. Although most attention has been paid to the use of these technologies in mitigating public health risks through disease vector modification or control, the applications to management of invasive species has not been overlooked [120].

In marine systems, a number of model and non-model organisms have already been explored as possible targets for CRISPR-based genomic modification, including such diverse taxa as diatoms, cnidarians, annelids, echinoderms, ascidians, and vertebrates [121]. These advances in marine species have generally lagged behind their terrestrial counterparts and have been directed primarily at development of tools for basic research. Nevertheless, early successes offer proofs of concept and suggest that genetic manipulation of wild marine populations may soon be an achievable goal. The potential promise of these tools for controlling highly damaging marine invasions is too great to ignore; however, the uncertainties associated with release of gene drive organisms in the wild demands that these technologies be explored with precaution and transparency [16,120,122]. This precaution was reflected in discussions at the meeting of the United Nations Convention on Biodiversity in December 2016, in which the proposal of a global moratorium on development of gene drives was ultimately rejected. It is further evidenced by the urgency of discussions aimed at erecting regulatory frameworks to guide research on gene drives and govern their application [123].

It is impossible to consider future development of genetic tools for managing marine invasions without addressing both the opportunities and the challenges presented by advanced biotechnologies. Besides general concerns associated with the effectiveness of gene drives for their intended application (e.g. evolutionary stability of transgenic elements and transmission efficiency), the management community needs to anticipate the risks of unintended consequences (e.g. uncontrolled spread of transgenes to non-target populations). As with traditional genetically-modified organisms, these risks may be further complicated by the broad connectivity of many marine systems and the unusual reproductive dynamics of many marine species. How effective would engineered limits on the transmission of gene drives be in the face of long-distance dispersal, either passive or active? How might sweepstakes recruitment and highly biased reproductive success shape the likelihood of successful population modification? Some of these concerns might be heightened in the case of widespread marine NIS. For instance, ongoing vector activity along well-established pathways of marine introduction almost certainly open routes of bi-directional population connectivity; to date no one has been concerned with the spread of invasive species *back* to their native ranges, but gene drives will certainly change that. Identification of appropriate targets for possible genetic biocontrol, institution of guidelines for secure development and laboratory testing of technologies, formal protocols for assessing risks of release into marine systems, establishment of standards for transparency and exchange of information between scientists and various stakeholders—these are all critical elements in the responsible assessment of these new tools, and should be approached intentionally and collaboratively within the context of the MSFD.

## 3. Conclusions

Following a century and a half of scientific documentation of marine bioinvasions in European seas, the development and implementation of management policies has been a slowly evolving and reactive process. The late realization that European seas are facing unprecedented rates of NIS introductions is reflected in the MSFD and in EU Regulation 1143/2014 [124], setting rules to prevent and manage the introduction and spread of invasive NIS in the EU—arguably the most important policy measures taken by the EU concerning marine bioinvasions. Yet, examination of the latest assessment of the Member States’ monitoring programs under the MSFD reveals that only 5% are related to NIS, and these “will require a clear acceleration to ensure proper coverage given the MSFD Deadlines for the update of marine strategies by 2018, and achieving Good Environmental Status by 2020” [13]. Improvements will be particularly important to achieve the specific goals for methodological standardization identified in the most recent decision of the European Commission [22]. A document prepared by the Joint Research Centre of the European Commission, providing technical guidance on monitoring for the MSFD, suggests “development for routine implementation of molecular-based methods” for NIS as the very last item under “possible research to implement at medium term or requiring modest investments” [15]. It is argued here instead that already available technologies offer critical assistance for obtaining needed monitoring data, and that significant investments of effort and funding may dramatically enhance future science-based solutions for management of marine bioinvasions, even in the near term. These technologies have the potential to considerably augment the existing monitoring toolkit, not only improving approaches for standard biodiversity assessments but also, in the case of metabarcoding and eDNA analysis, enabling more comprehensive analysis of ecosystem-wide impacts of marine invasions.

Molecular technologies advance rapidly. There is some danger that this observation might encourage complacency; the temptation is to assume that managers need only wait, and a molecular tool appropriate for their needs will be forthcoming. Though there may be some truth in the capacity of the research community to develop useful tools in the absence of external guidance, there is also no doubt that the deliberate, collaborative exploration of molecular technologies is likely to generate many more such tools, and more rapidly, than would otherwise be possible. Early engagement of stakeholders can be critically important not only for the development of novel molecular tools, but to their ultimate acceptance among end-users [21,49]. Transparency and collaboration are powerful antidotes to skepticism, and we strongly encourage rapid adoption of proactive, cooperative approaches to molecular tool development with the explicit aim of improving the monitoring capacities of MSFD Member States.

## Figures and Tables

**Table 1:** Applications of genetic technologies to NIS surveillance objectives within the context of the MSFD. Accessibility to non-specialist is assessed based on currently available technology.

NIS survey objective	Most relevant MSFD-related objective	Applicable technology	Specific strength (s)	Accessibility to non-specialist	Upstream research needed to increase relevance and accessibility
Validate a specific NIS identification first based on morphology	early detection of NIS	Single specimen collection Standard (Sanger) sequencing	Standard and widely available laboratory method	High	Improve DNA reference data for many European NIS
Targeted (species-specific) surveys	early detection of NIS; Trends in NIS distribution	Bulk DNA or eDNA combined with species-specific probes (e.g. qPCR or other targeted approach)	Increasingly standardized and available molecular methods; Cost effective for surveying a large number of localities/samples	Medium	Improve reference data when lacking; standardized sampling protocols; design of sensitive and specific probes; improve models to infer population distribution from survey results
Targeted (taxon- or group- specific inventories (e.g. fish, protists))	Trends in NIS distribution; Impacts to native biodiversity	Bulk DNA or eDNA combined with HTS using dedicated (taxa-specific) primers and/or dedicated reference database	Cost-effective for processing a large number of samples; potential for broad taxonomic coverage	Low	Improve reference data; standardization of bioinformatic workflows; improve inferences of relative abundance from HTS data; develop user-friendly tools
Non-targeted global inventories	Trends in NIS distribution; Impacts to native biodiversity; broad shifts in ecosystem structure and function	Bulk DNA or eDNA combined with HTS using “universal” primers and databases	Cost-effective for processing a large number of samples; potential for broad taxonomic coverage; surveillance of non-traditional taxa (e.g. meiofauna or microbial communities)	Low	Improve reference data; standardization of bioinformatic workflows; improve inferences of relative abundance from HTS data; develop user-friendly tools

## Glossary of terms. Terms are provided in alphabetical order, not in the order in which they appear in the text

Bulk DNA: DNA obtained from community samples targeting particular organisms, such as from plankton collected with a plankton tow or large-size organisms scraped from rocks or collected in grabs
CRISPR/Cas9: A genome-editing tool (derived from a bacterial “immune system” designed to confer resistance to foreign genetic elements) that allows researchers to easily modify a portion of a target organism’s genome by adding, removing, or altering DNA sequence. This technique can be used in the development of gene drives (see below)
DNA barcoding: Certain small regions of the genome evolve in such a way that sequence variation in those regions is very low within species but relatively high between species. This allows sequence from these regions—the “barcode loci”—to be used to determine species identity of a specimen with some confidence, assuming the existence of suitable reference data (see definition below)
Environmental DNA (eDNA): Extra-organismal DNA molecules are ubiquitous in the environment in shed cellular and extracellular material, released from skin, mucous, saliva, sperm, secretions, eggs, faeces, urine, blood, leaves, fruit, pollen, and rotting bodies. Such DNA is collectively referred to as eDNA, and its persistence in aquatic systems depends on temperature, flow, pH etc. eDNA comprises all genetic material occurring in bulk environmental samples even absent the organism and is often more easily captured than target organisms. It can be used to infer patterns of population distribution
Gene drive: A genomic modification of a study organism that results in the inheritance of a particular genetic element at rates far higher than those expected from Mendelian inheritance. Gene drives can cause the rapid spread of a genetic element throughout a sexually reproducing population, even if there is a selective disadvantage of that element.
High throughput sequencing: (HTS; formerly referred to as next generation sequencing, NGS). Technologies that generate large volumes of sequence data (millions of individual sequences) rapidly and inexpensively through parallelization of large numbers of sequencing reactions. While initial investments in equipment can be high, subsequent cost per unit sequence is extremely low
Integrative taxonomy: The science of characterizing, classifying, and naming taxa based on a multidisciplinary approach incorporating morphological, ecological, molecular genetics, and evolutionary insights. It offers a scientific approach aiming at proposing stable and testable species hypotheses based on multiple lines of evidence
Metabarcoding: Instead of generating barcode sequence from each individual species found in a community, a pool of barcode sequences can be generated by extracting bulk DNA from that community and then using “universal” primers and high throughput sequencing to generate barcodes from all the organisms present in the sample. Bioinformatic analysis of these sequence pools, using available reference data, allows assignment of barcode sequences to likely species, revealing the taxonomic diversity in the sample
Metagenomics: The generation and analysis of sequence data derived broadly from throughout the genomes of organisms (or from whole genomes when small, e.g. bacteria) present in an environmental sample. Contrast with metabarcoding, which focuses on a single genomic locus
Non-target surveillance: Surveillance of biodiversity conducted without *a priori* identification of a surveillance target. Typically conducted using metabarcoding approaches, often in coordination with eDNA sampling
Operational taxonomic unit (OTU): An operational definition used to classify groups of closely related individuals based on sequence data. Typically, OTUs are defined based on a threshold of sequence similarity (e.g. 97% similarity across a particular barcode sequence)
Reference database: To assign a species name to a specimen barcode, it is necessary to compare that barcode to an existing sequence that has been attached to a species name by a competent expert. Reference databases provide public access to such sequences. Ideally, sequences in reference databases have been subjected to rigorous quality control for sequence quality and taxonomic accuracy
Site occupancy-detection modelling (SODM): Models that estimate the population distribution of a monitored species across sites using observed patterns of detection of that species
Targeted surveillance: Surveillance directed at detecting the presence of a specific target taxon. Typically takes advantage of species-specific molecular genetic probes (frequently through PCR or qPCR) to recognize the presence of the target in the sample
